# Familial Atypical Hemolytic Uremic Syndrome: A Review of Its Genetic and Clinical Aspects

**DOI:** 10.1155/2012/370426

**Published:** 2012-11-08

**Authors:** Fengxiao Bu, Nicolo Borsa, Ardissino Gianluigi, Richard J. H. Smith

**Affiliations:** ^1^Interdepartmental PhD Program in Genetics, University of Iowa, Iowa City, IA52242, USA; ^2^Division of Nephrology, Department of Internal Medicine, Carver College of Medicine, University of Iowa, 5270 CBRB, Iowa City, IA 52242, USA; ^3^Laboratory of Molecular Genetics, Fondazione IRCCS Cà Granda Ospedale Maggiore Policlinico, Milan 20122, Italy; ^4^Center for HUS Control, Fondazione IRCCS Cà Granda Ospedale Maggiore Policlinico, Milan 20122, Italy

## Abstract

Atypical hemolytic uremic syndrome (aHUS) is a rare renal disease (two per one million in the USA) characterized by microangiopathic hemolytic anemia, thrombocytopenia, and acute renal failure. Both sporadic (80% of cases) and familial (20% of cases) forms are recognized. The study of familial aHUS has implicated genetic variation in multiple genes in the complement system in disease pathogenesis, helping to define the mechanism whereby complement dysregulation at the cell surface level leads to both sporadic and familial disease. This understanding has culminated in the use of Eculizumab as first-line therapy in disease treatment, significantly changing the care and prognosis of affected patients. However, even with this bright outlook, major challenges remain to understand the complexity of aHUS at the genetic level. It is possible that a more detailed picture of aHUS can be translated to an improved understanding of disease penetrance, which is highly variable, and response to therapy, both in the short and long terms.

## 1. Introduction

Hemolytic uremic syndrome (HUS) is a rare disease characterized by microangiopathic hemolytic anemia, thrombocytopenia, and acute renal failure. It is most frequently caused by infections of Shiga-like toxin producing bacteria, such as *Escherichia coli* strain O157 : H7, O111 : H8, O103 : H2, O123, and O26 [1]. In approximately 10% of HUS cases, there is no association with Shiga-like toxin. These cases are classified as atypical HUS (aHUS) and occur with an incidence of about 2 per million in the USA [1, 2]. aHUS patients have a poorer prognosis than those with typical HUS, with acute phase aHUS mortality of about 8% [3, 4], and with 50%–80% of aHUS patients progressing to end-stage renal failure [1]. However, it is important to note that epidemiological outcomes data are relatively out of date because of the development of better diagnostic, treatment, and management strategies.

Atypical HUS can be classified as sporadic or familial. Familial aHUS is defined as the presence of aHUS in at least two members of the same family with diagnoses at least 6 months apart [1, 3, 5]. It accounts for less than 20% of aHUS cases [3]. In familial aHUS (and also sporadic aHUS), genetic (e.g., gene mutations, rare variants, and risk haplotypes) and acquired abnormalities (e.g., autoantibodies against factor H) are found in ~70% of patients [6]. Gene mutations are usually found in complement genes, such as factor H (*CFH*), factor I (*CFI*), factor B (*CFB*), complement component 3 (*C3*), and membrane cofactor protein (*MCP* or *CD46*). Evidence from familial studies indicates a high rate of incomplete penetrance, with about 50% of carriers of *CFH* or *MCP* aHUS-associated variants not developing disease [7]. The reasons underlying incomplete penetrance are unclear, although it is recognized that multiple predisposing genetic variants and risk haplotypes exist which may be relevant to disease onset in the face of environmental triggers such as pregnancy, viral infection, cancer, organ transplantation, and the use of certain drugs [8, 9]. 

In this paper, we focus on familial aHUS. Over the past 20 years, dozens of aHUS pedigrees have been reported, clarifying the underlying mechanisms of both familial and sporadic aHUS. In followed sections, we will discuss the complement system and aHUS, genetic abnormalities identified in familial studies, factors associated with incomplete penetrance, and current methods of diagnosis and treatment. 

## 2. The Complement System 

**Figure 1:**
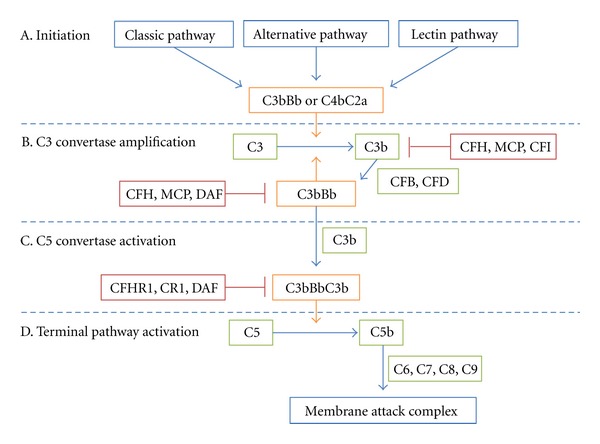
The complement system and its regulators. The complement system has four main steps. (A) Classical, alternative, or lectin pathway activation produces C3 convertases (C3bBb or C4bC2a) to initiate the complement cascade. (B) C3 convertase cleaves C3 into C3a and C3b. CFB binds to C3b and is cleaved by CFD into Bb, forming a new C3 convertase, C3bBb. This amplification step is tightly controlled by multiple regulators of complement (e.g., CFH, MCP, DAF, and CFI). (C) Once C3 convertase amplification is allowed to proceed, additional C3b is generated, ultimately forming C5 convertase, C3BbC3b. (D) C5 convertase cleaves C5 into C5b, which recruits C6, 7, 8, and 9 to form the membrane attack complex.

The complement system is an essential component of the innate immunity (Figure 1). Its four major steps are: (1) the initiation of the complement cascade; (2) C3 convertase activation and amplification; (3) C5 convertase activation; (4) terminal pathway activation [10]. Initiation of the complement cascade occurs through three pathways: the classical pathway [11, 12], the lectin pathway [13, 14], and the alternative pathway [15, 16]. Once activated, C3 convertases are formed (the alternative pathway forms C3bBb, and the classical pathway or the lectin pathway forms C4bC2a), which cleave C3 to C3a and C3b. C3b can indiscriminately bind to surfaces of microbes and host cells [17, 18]. On the surface of microbes or modified host cells, C3b and factor B form more C3 convertases, which produce more C3b. This amplification process exponentially increases the amount of C3b and C3 convertases. 

On the surface of intact host cells, in contrast, C3b deposition and C3 convertase amplification are prevented by complement regulators. Regulators distribute in the fluid phase (CFH, CFHR1, CFP, etc.) and on cell surfaces (CR1, MCP, DAF, etc.) to control complement activity through two major mechanisms: decay acceleration activity and cofactor activity [10]. CFH, for example, acts as a cofactor with CFI to cleave C3b to an inactive form, iC3b; has the decay acceleration activity, which promotes the decay of the C3 convertase [19, 20] and competes with CFB for binding to C3b. If C3 convertase amplification is allowed to proceed unchecked, additional C3b binds to C3 convertases to generate C5 convertases (C3bBbC3b or C4bC2aC3b) [21]. C5 convertases cleave C5 to C5a and C5b to initiate the terminal pathway and form terminal complement complexes with C6, C7, C8, and C9 to lyse target cells [22, 23]. 

**Figure 2:**
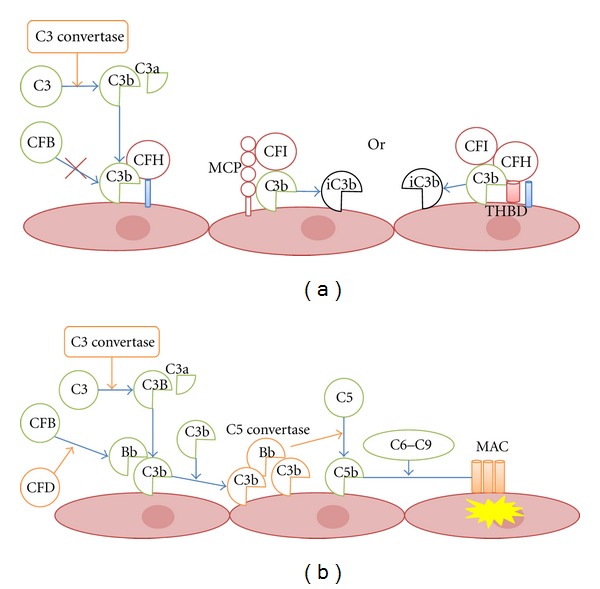
Regulation and dysregulation of complement activity on host cells. C3b is generated by the classical, lectin, or alternative pathways. (a) To protect normal host cells, C3b is inactivated by membrane regulators, such as factor H (CFH) and membrane cofactor protein (MCP). Factor I (CFI) cleaves C3b into iC3b and other C3 degradation products with the activity of cofactor regulators. (b) If genetic and/or environmental risk factors reduce the efficiency of membrane complement regulators, C3 convertase (C3bBb) can accumulate on cell surfaces, creating a C3b amplification loop. Formation of C5 convertase (C3bBbC3b) triggers C5 cleavage into C5b, which interacts with C6, C7, C8, and C9 to generate membrane attack complex (MAC) leading to cell damage.

Mutations of complement genes can either change expression level or disrupt protein function.  Figure 2 shows a model of dysregulation of complement regulators inducing aHUS. Mutations in complement genes impair the regulation of C3b on host cells, leading to formation of membrane attack complex and host cell damage. Most genetic abnormalities in aHUS patients are found in complement membrane regulators and C3 convertases. Multiple genetic and environmental risk factors are believed necessary to develop disease; however, relatively little is known about how environmental triggers affect homeostasis of complement system in the face of predisposing genetic variants in complement genes [8]. It is also unclear whether genetic variants in complement gene increase susceptibility to typical HUS [24, 25]. 

## 3. Genetic Abnormality in Family Cases

The term “familial aHUS” is used to describe families in which two or more persons develop aHUS at different times without exposure to common triggering infectious agents, or when disease-causing mutations are identified in one of the genes (discussed next) known to be associated with aHUS irrespective of familial history [1]. Thus, genetic aHUS can be multiplex (two or more affected family members) or simplex (a single occurrence in a family). Since simplex cases develop in patients who do not have a family history of disease, these cases are also referred to as sporadic [1]. 

The first familial aHUS case was reported in concordant monozygotic twins in 1965 [26]. Since that report, autosomal dominant and recessive familial aHUS has been reported. Familial studies have revealed important genetic factors contributing to aHUS, including mutations in *CFH*, *CFHR3*, *MCP*, *CFI*, *CFB*, and *C3* (Table 1). Most of these mutations impair protein function, causing dysregulation of the complement pathway once it has been activated. Interestingly, some genes implicated in sporadic aHUS, such as *THBD *[5], have not been associated with familial cases to date. It is reasonable to expect that comprehensive genetic screening of genes in the complement and coagulation pathways will identify variants in additional genes that impact disease penetrance, consistent with aHUS being a complex genetic disease. 

### 3.1. *CFH* and *CFHR* Mutations

Complement factor H, encoded by *CFH* gene, is an essential inhibitor of C3 convertase and a central regulator of the complement alternative pathway. It is produced by liver as a soluble protein but can attach to and act on cell surfaces. CFH protein contains 20 repetitive units of about 60 amino acids named short consensus repeats (SCRs; also known as complement control protein (CCP) repeats or Sushi domains) [27]. N-terminal SCRs regulate binding to C3b, while C-terminal SCRs facilitate cell-surface binding and regulation. CFH regulates the complement system through three mechanisms: (1) inhibiting the assembly of C3 convertase by competitive binding to C3b, (2) accelerating the decay of C3 convertase, and (3) acting as a cofactor in the cleavage and degradation of C3b by CFI [27].

*CFH* is the most thoroughly studied gene in aHUS. Mutations associated with aHUS were first identified in *CFH* by a familial genetic study in 1998 when Warwicker and colleagues conducted linkage analysis in three aHUS families and mapped the aHUS risk region to a 26-cM interval on chr 1q32 with a lod score of 3.94. The linked region includes the *CFH* and *CFHR* genes, and in sequencing *CFH*, a heterozygous c.3716C>G variant changing an arginine to glycine was found to cosegregate with disease in family 2. In family 3, a *CFH* deletion, c.145_148delAGAA, was found [28]. 

Numerous missense transversion and transition variants in *CFH* have now been associated with aHUS through familial studies [29–34]. Typically, affected patients are heterozygous for these changes, which predominantly occur in the C-terminal SCRs 19 and 20. Since these SCRs are essential for cell-surface attachment, this finding suggests that membrane dysregulation of the complement system is critical to the pathogenesis of aHUS.

Based on available studies, we estimate that penetrance of *CFH *mutations ranges from 12.5% to 100% (Table 1). It is remarkable that one *CFH* rare variant, rs121913059 (c.3701C>T or p.R1210C), has been reported in five families from three familial studies [29, 30, 32]. This variant decreases CFH binding to C3b, heparin, and endothelial cells, and leads to a positive sheep erythrocyte hemolytic assay [35]. The Y402H variant (rs1061170, c.1204T>C) of *CFH*, notable for its association with age-related macular degeneration [36], dense deposit disease, and C3 glomerulonephritis [37], has not been associated with aHUS. However, Hakobyan et al. have reported low expression of the CFH-H402 allele in association with other known aHUS variants in two aHUS families, suggesting that in some instances the CFH-H402 allele may contribute to the aHUS phenotype [32]. 

In addition to *CFH*, *CFHR3* has been linked with familial aHUS. The *CFH*-related genes (*CFHR1*, *CFHR2*, *CFHR3*, *CFHR4,* and *CFHR5*) localize next to *CFH* and share many of the functional properties of *CFH*. A recent study has reported a hybrid *CFH/CFHR3* gene caused by a microhomology-mediated deletion that is associated with familial aHUS. The transcript product of the hybrid gene contains 24 SCRs with SCRs 1–19 deriving from *CFH* and SCRs 20–24 deriving from *CFHR3*. The hybrid protein shows normal fluid-phase activity but loses complement regulation on cell surfaces [38]. 

### 3.2. *MCP* Mutations

*MCP* (*CD46*) encodes membrane cofactor protein, which acts as a cofactor for CFI to regulate complement activity by cleaving C3b and C4b deposited on the surface of host cells. MCP is a transmembrane protein with four N-terminal extracellular Sushi domains, a transmembrane domain, and a C-terminal cytoplasmic tail. Sushi domains 3 and 4 are responsible for complement regulation [39]. 

*MCP* is well studied in aHUS. In 2003, Richards et al. [40] first reported mutations of *MCP* in aHUS families. Two mutations were found in three families—a 6 bp deletion (p.237_238delDS) and c.822T>C (p.S206P, rs121909589). The c.822T>C mutation causes an amino acid change of serine to proline and leads to a significant reduction of C3b binding. Soon afterwards, Noris et al. reported another aHUS family carrying a 5 bp deletion in *MCP* gene, which causes a premature stop codon in the fourth Sushi domain [41]. Expression analysis showed around 50% reduction in MCP as compared to healthy controls. Subsequently, several more *MCP* mutations have been identified in aHUS families [7, 33, 42–44]. 

Studies indicate that MCP mutations account for up to 15% of aHUS patients [7, 33, 42]. Although the majority of MCP mutations are heterozygous (~75%), some homozygous or compound heterozygous mutations in MCP have been reported [45]. MCP mutations are defined as (a) type I (~75%) if they reduce expression on cell surface and (b) type II (~25%) if expression is normal but complement regulatory activity is impaired [46, 47].

The *MCP* ggaac haplotype formed by c.−652A>G (rs2796267), c.−366A>G (rs2796268), c.IVS9−78G>A (rs1962149), c.IVS12+638G>A (rs859705), and c.4070T>C (rs7144), is associated with aHUS in both sporadic and familial cases [43, 48, 49]. Further studies are needed to determine the functional or expression differences between *MCP ggaac* and normal haplotypes.

### 3.3. *C3* Mutations

Complement component C3 is the keystone in the complement system. It undergoes spontaneous hydrolysis and is also cleaved by C3 convertase to C3b and C3a. C3b interacts with CFB to form C3bB, which is then cleaved to C3bBb by CFD forming the C3 convertase. Additional C3b leads to the formation of C5 convertase, which activates the terminal pathway. C3 products are key ligands for multiple complement regulators, including CFH and MCP. Theoretically, mutations influencing C3 binding ability or other functions could disrupt complement regulation and contribute to the development of aHUS.

Reported C3 mutations are heterozygous and localized on both the beta and alpha chains. In 2008, Frémeaux-Bacchi et al. first reported nine mutations of *C3* in 14 patients from 11 families, including a p.R570W mutation in a very large family. Five of the nine identified mutations (p.R570Q, p.R570W, p.A1072V, p.D1093N, and p.Q1139K) reduce ligand binding to MCP, making the mutant convertase resistant to cleavage by CFI thus impairing complement regulation of C3 convertase amplification on cell membranes [50]. Lhotta et al. have also reported a large Austrian family carrying the p.R570Q C3 mutation. In their study, they showed reduced or borderline C3 levels in mutation carriers [51]. Recently, another familial C3 mutation, V1636A, has been identified to cause increased affinity of CFB for C3b [52]. 

### 3.4. *CFB* Mutations

Complement factor B, a key component of C3 convertase (C3bBb), contains three Sushi domains, a vWFA domain, and a peptidase S1 domain. It is cleaved by CFD into Ba and Bb. Bb is a serine protease, which binds to C3b to generate the C3 convertase.

In 2007, Goicoechea de Jorge et al. reported a CFB gene mutation in an aHUS family with seven patients [53]. Sequence analysis and functional studies indicated that the missense mutation, c.858C>G (p.F286L), in the vWFA domain, caused more rapid formation and a higher level of C3 convertase. Penetrance was incomplete with seven of 11 mutation carriers developing aHUS. Interestingly, the *MCP* ggaac risk haplotype was found only in patients and one young carrier who is probably still at risk for disease, suggesting that the effects of the CFB variant are modulated by variants in other complement gene.

### 3.5. *CFI* Mutations

Complement factor I is an inhibitory regulator of complement system. It cleaves C3b or C4b with the presence of cofactors, such as CFH and MCP, to iC3b, which is cleaved to smaller C3 degradation products. Defects in *CFI* cause multiple complement-related diseases, including aHUS and CFI deficiency (OMIM: 610984), a disease characterized by recurrent infections and glomerulonephritis in some patients [54].

Most *CFI* mutations have been found in sporadic aHUS cases. These mutations either interrupt cofactor activity or impact the expression level of CFI [55]. In familial aHUS studies, CFI mutations have been reported in three pedigrees (Table 1) [33, 43, 52]. In a Spanish family, a heterozygous 2 bp insertion within the coding region of *CFI* has been identified to cause a premature stop codon, p.T538X, which reduces plasma levels of CFI by 50%. A missense mutation in *MCP* (c.598C>T) and the *MCP* ggaac risk haplotype were also identified in this family, with all patients carrying all three genetic risk factors. Nine unaffected persons carry only one or two genetic risk factors, suggesting that it is the combination of mutations and the risk haplotype that are critical to the development of aHUS [43].

### 3.6. Combined Mutations and Incomplete Penetrance of Familial aHUS

Multiple familial studies have reported that it is the combination of complement gene mutations that contributes to aHUS [29, 30, 32, 42, 43, 52, 53]. For instance, in a study by Sartz et al., four mutations, one each in *C3*, *MCP*, *CFI,* and *CFH*, were found in two patients from a single family [52]. The *C3* mutation, p.V1636A, increases the affinity for CFB and C3 convertase; the *MCP* mutation, p.A304V, increases the activation of the alternative pathway on cell surfaces; and although the functional significance of the *CFI* mutation (c.IVS12+5) and the *CFH* mutation (p.Q950H) is unknown, they have been reported in other aHUS cases [52]. The aggregate data suggest that accumulated dysregulation by combined mutations impairs the complement system and leads to disease [29, 30, 32, 42, 43, 52, 53]. It is unknown whether other complement factors, such as *THBD*, *CR1*, *C5*-*C9*, and *DAF*, contain risk variants that contribute to the mutation/variant load in aHUS. 

Incomplete penetrance is widely observed, with the estimated penetrance of aHUS in mutation carriers being about 50~60% [7, 8]. Within families, affected persons may also show different symptoms and onset ages [56]. These findings strongly suggest that most aHUS-associated genetic variants predispose to rather than cause the disease. However, the genetic picture is incomplete as most studies have focused on only the common complement genes in a disease where rare genetic variants in other complement genes and genes in other pathways are likely to be contributory to the phenotype. Importantly, the effect of common variants is probably marginal as demonstrated by Ermini and colleagues who tested 501 SNPs in 47 complement genes in 220 aHUS patients and 549 controls and found disease associations for only *CFH*, *MCP,* and the *CFHRs* [49]. However, until a comprehensive rare variant screen is completed, it will remain very difficult to calculate disease risk for persons in aHUS families. 

## 4. Diagnosis and Treatment

aHUS is clinically characterized by microangiopathic hemolytic anaemia (low hemoglobin, high lactic acid dehydrogenase, undetectable or low haptoglobin, presence of schistocytes in the peripheral blood smear, and negative Coombs test), thrombocytopenia (platelets < 150000/mm^3^ or a documented rapid decrease), and acute kidney injury (AKI) (hematuria, proteinuria, and/or reduced renal function). However, as a systemic disease, aHUS can affect the endothelia of any organ, and extrarenal manifestations including involvement of the central nervous system, liver, heart, pancreas, and skin, are observed in as many as 20% of patients [3, 4]. These additional sites of involvement can blur the distinction between aHUS and other primary thrombotic microangiopathies (TMAs), such as STEC-associated HUS, thrombotic thrombocytopenic purpura (TTP), HELLP syndrome (hemolytic anemia, elevated liver enzymes, and low platelets), and transcyanocobalamin deficiency, or TMAs secondary to malignant hypertension, catastrophic antiphospholipid syndrome, or disseminated intravascular coagulation. 

The treatment of aHUS is based on two main strategies: supportive treatment and cause-specific treatment. The former is focused on careful fluid, electrolyte, acid-base, and nutritional management, with the use of blood transfusion, antihypertensive medications, and/or dialysis, as needed. Cause-specific treatment includes plasma therapy provided by either plasma infusion (fresh frozen plasma, 20–40 mL/kg/day if the patient is not volume overloaded) or by high volume plasma exchange with fresh frozen plasma (150% of plasma volume daily or every other day until clinical remission). A recent impressive improvement in the management of aHUS has been reported with the use of the anti-C5 monoclonal antibody, Eculizumab, which binds to C5 thereby preventing activation of the terminal complement cascade. This relatively new (since 2009) treatment is continued until stable clinical remission. Whether life-long or recurrence-specific treatment is necessary and how genetics may or may not impact care of persons on Eculizumab have not been determined.

## 5. Transplantation

Only recently has renal transplantation become the treatment of choice for patients in end stage renal disease on chronic dialysis for aHUS. Until the availability of Eculizumab, transplantation was associated with a 40%–80% risk for disease recurrence [3, 57–59]. The notable exception was aHUS patients with *MCP* mutations since MCP is expressed on the renal endothelia and not in the fluid phase. Eculizumab has become a key resource for preventing recurrence following kidney transplantation and for rescue therapy in case of disease recurrence. Combined liver-kidney transplant with preemptive and perioperative plasma therapy [60], although successfully used in the very recent past, in the Eculizumab era no longer appears to be first-line treatment. Transplantation with a living-related donor is not recommended given our current incomplete understanding of the genetics of aHUS.

## 6. Conclusion

Over the past two decades, studies of familial aHUS have greatly increased our understanding of this disease. The identification of genetic variants in complement genes has defined the mechanism whereby complement dysregulation at the cell surface level leads to disease. This understanding has culminated in the use of Eculizumab as first-line therapy in disease treatment, significantly changing the care and prognosis of affected patients. However, even with this bright outlook, major challenges remain to understand the complexity of aHUS at the genetic level. It is possible that a more detailed picture of aHUS can be translated into patient-specific short- and long-term therapy with Eculizumab and/or other anticomplement drugs in the developmental pipeline. 

## Figures and Tables

**Table 1:** Reported gene mutations and risk haplotypes in aHUS pedigrees^#^.

Author	Year	Population	Size/carrier/affected	Gene: variant or haplotype	Risk genotype	Penetrance rate	SNP rs ID	MAF
Warwicker et al. [28]	1998		51/3/11*	*CFH*: c.3716C>G (p.R1197G)	C/G		rs121913051	
				*CFH*: c.145_148delAGAA	het			
Ying et al. [31]	1999	Bedouin-Arab	55/?/11**	*CFH*: c.3645C>T (p.S1191L)	T/T		rs460897	29.35%
Richards et al. [40]	2003	Belgian	8/0/3	*MCP*: p.237_238delDS	het	100.0%		
		German	4/1/2	*MCP*: c.822T>C (p.S206P)	T/C	66.7%	rs121909589	
		Turkish	4/2/2	*MCP*: c.822T>C (p.S206P)	C/C	50.0%	rs121909589	
Caprioli et al. [30]	2003		13/1/2	*CFH*: c.1494_1496delAAA	het	66.7%		
			5/1/2	*CFH*: c.3620T>A (p.Y1183R)	T/A	66.7%		
			10/5/2	*CFH*: c.3654G>A (p.G1194D)	G/A	28.6%		
			6/1/3	*CFH*: c.3701C>T (p.R1210C)	C/T	75.0%	rs121913059	0.02%
			37/?/10*	*CFH*: c.3579A>T	A/T			
				*CFH*: a 24-bp deletion in SCR20	het			
			9/2/3	*CFH*: c.3717G>A (p.R1215Q)	G/A	60.0%		
Noris et al. [41]	2003	White	4/1/2	*MCP*: c.843_844delAC	het	66.7%		
Frémeaux-Bacchi et al. [42]	2006	White	5/0/3	*MCP*: p.G162R	het	100.0%		
			4/0/2*	*MCP*: p.Y155D	het	100.0%		
				*MCP*: c.IVS7-2A>G	het	100.0%		
Esparza-Gordillo et al. [43]	2006	Spanish	24/11/2*	*MCP*: c.598C>T(p. P165S)	C/T	28.6%		
				*MCP*: MCPggaac***	het	33.3%		
				*CFI*: c.1610insAT(p.T538X)	het	28.6%		
Caprioli et al. [7]	2006	Sardinian	8/4/3*	*MCP*: D1S2735, D1S2796, IVS1-1G>C, ExV(SCR3), Ex XII, D1S2692	2,1,+,A,T,8	42.9%		
				*MCP*: c.IVS1-1G>C	G/C	42.9%		
			8/1/5*	*MCP*: c.218C>T	C/T	85.7%		
				*MCP*: c.147G>A	G/A	85.7%		
			5/2/2	*MCP*: c.843_844delAC	het	50%		
			21/5/2	*MCP*: c.768T>G	T/G	28.6%		
Goicoechea de Jorge et al. [53]	2007	Spanish	32/4/7*	*CFB*: c.858C>G (p.F286L)	C/G	64.0%		
				*MCP*: MCPggaac	het	87.5%		
Frémeaux-Bacchi et al. [50]	2008		54/?/6	*C3*: p.R570W	C/T			
Martinez-Barricarte et al. [29]	2008		6/1/3	*CFH*: p.R1210C	C/T	12.5%	rs121913059	0.02%
Lhotta et al. [51]	2009	Austrian	61/9/4	*C3*: c.1775G>A (p.R570Q)	G/A	10.0%	rs121909583	
Habibi et al. [34]	2010	Tunisian	33/10/6	*CFH*: c.3767_3771delTAGA	hom	37.5%		
Sullivan et al. [33]	2010		6/3/2	*CFH*: c.3007G>T (p.W978C)	G/T	40.0%		
			5/2/2	*CFH*: c.3619G>T (p.R1182S)	G/T	50.0%		
			4/1/2	*MCP*: c.404delG (p.G135VfsX13)	het	66.7%		
			3/?/2	*CFI*: c.491A>T (p.D164V)	A/T			
Hakobyan et al. [32]	2010		15/3/4	*CFH*: p.C853R, H402	het+H	33.3%		
Provaznikova et al. [44]	2012		3/0/2	*MCP*: c.1148C>T	C/T			
			2/0/2	*MCP*: c.404G>A	G/A			
			2/0/2	*MCP*: c.350insA	het			
			7/2/2	*MCP*: c.2T>A	T/A	50.0%		
Sartz et al. [52]	2012		20/2/4*	*C3*: c.4973T>C (p.V1636A)	T/C	100.0%		
				*CFI*: c.IVS12+5G>T	G/T	100.0%		
				*MCP*: c.1058C>T (p.A304V)	C/T	50.0%		
				*CFH*: c.2850G>T (p.Q950H)	G/T	50.0%		
Francis et al. [38]	2012		35/4/3	*CFH/CFHR3* hybrid	het	42.9%		

^#^Pedigrees are included if at least two family members were diagnosed with aHUS at least 6 months apart.

*More than one mutation identified within the family.

**? presents undetermined number.

***The risk haplotype *MCP* ggaac is formed by rs2796267, rs2796268, rs1962149, rs859705, and rs7144.
